# In Silico Analysis of Glucose Oxidase from *Aspergillus niger*: Potential Cysteine Mutation Sites for Enhancing Protein Stability

**DOI:** 10.3390/bioengineering8110188

**Published:** 2021-11-19

**Authors:** Sirawit Ittisoponpisan, Itthipon Jeerapan

**Affiliations:** 1 Center for Genomics and Bioinformatics Research, Division of Biological Science, Faculty of Science, Prince of Songkla University, Hat Yai, Songkhla 90110, Thailand; 2 Center of Excellence for Trace Analysis and Biosensor, Prince of Songkla University, Hat Yai, Songkhla 90110, Thailand; 3 Division of Physical Science, Faculty of Science, Prince of Songkla University, Hat Yai, Songkhla 90110, Thailand; 4 Center of Excellence for Innovation in Chemistry, Faculty of Science, Prince of Songkla University, Hat Yai, Songkhla 90110, Thailand

**Keywords:** glucose oxidase, glucose, stability, protein engineering, disulfide bond, mutagenesis, *Aspergillus niger*, in silico study

## Abstract

Glucose oxidase (GOx) holds considerable advantages for various applications. Nevertheless, the thermal instability of the enzyme remains a grand challenge, impeding the success in applications outside the well-controlled laboratories, particularly in practical bioelectronics. Many strategies to modify GOx to achieve better thermal stability have been proposed. However, modification of this enzyme by adding extra disulfide bonds is yet to be explored. This work describes the in silico bioengineering of GOx from *Aspergillus niger* by judiciously analyzing characteristics of disulfide bonds found in the Top8000 protein database, then scanning for amino acid residue pairs that are suitable to be replaced with cysteines in order to establish disulfide bonds. Next, we predicted and assessed the mutant GOx models in terms of disulfide bond quality (bond length and α angles), functional impact by means of residue conservation, and structural impact as indicated by Gibbs free energy. We found eight putative residue pairs that can be engineered to form disulfide bonds. Five of these are located in less conserved regions and, therefore, are unlikely to have a deleterious impact on functionality. Finally, two mutations, Pro149Cys and His158Cys, showed potential for stabilizing the protein structure as confirmed by a structure-based stability analysis tool. The findings in this study highlight the opportunity of using disulfide bond modification as a new alternative technique to enhance the thermal stability of GOx.

## 1. Introduction

Glucose oxidase (GOx; Enzyme Commission Number: 1.1.3.4) is an enzyme found in several natural sources, including insects, fruits, and fungi. GOx is widely extracted from *Aspergillus niger*, a haploid filamentous fungus, which can be abundant in warm environments, in field conditions, and in stored foods [1,2]. GOx from *A. niger* is of great importance, particularly in trade. Therefore, this commercially available GOx is one of the most common biomolecular structures used in research and industries. GOx is a viable oxidoreductase containing a flavoprotein capable of the biocatalysis of the oxidation of β-D-glucose to D-glucono-1,5-lactone and hydrogen peroxide (H_2_O_2_). Spontaneously, the gluconolactone molecule can be transformed into gluconic acid. The GOx-based catalysis also uses oxygen as an electron acceptor. As such an important bioreaction involves glucose, oxygen, H_2_O_2_, and electrons, GOx has been applied to several domains, ranging from food and pharmaceutical sciences to biosensors and bioelectronics (such as glucose biosensors and enzymatic biofuel cells) [3,4]. Nevertheless, a formidable challenge pertaining to long-term stability, particularly in fluctuating temperature environments, impedes the progress of GOx-based biotechnology. For example, the new trend of wearable GOx-based bioelectronics has been challenged by enzyme stability because, in real scenarios, the enzyme embedded in the device has to be stored and operated in noncontrolled environments [5]. More importantly, GOx-based devices, e.g., implantable enzymatic bioelectronics, are also required to be sanitized. The process of device sanitization usually comes by means of heat sterilization [6]. Hence, finding a strategy to enhance the thermal stability of the enzyme is crucial for the more effective use of GOx.

Experimental efforts to tailor the external protective microenvironment of the immobilized enzyme and the efforts on random mutagenesis to engineer the enzymatic structure itself have been devoted to enhancing the thermal stability when using GOx [7,8,9]. To effectively find an alternative way, the modification of enzymes by protein-structure engineering can judiciously tailor protein behaviors and secure the protein structure. This approach would accurately predict the optimal structure by leveraging computational bioengineering to tune the stability, thus narrowing down the finest design. This would guide how to tune inherent structures of the protein in a high-throughput and efficient way, allowing the robust structure while the enzyme is used in harsh surroundings.

In silico prediction is often seen as the first step for protein engineering. To enhance protein thermal stability, modeling extra disulfide bonds to a protein structure is one of the possible mutagenesis strategies. Other common strategies include introducing hydrophobic amino acids to make a more hydrophobic surface or create hydrophobic interactions, enhancing overall hydrophobicity [10], and adding extra bondings between side chains of amino acids (such as hydrogen bonds and salt bridges) [11]. Interestingly, it was found that many stabilizing amino acid substitutions tend to be hydrophobic, and that also led to many computational tools for in silico protein design favoring hydrophobic substitution [12,13]. Despite its popularity, enhancing hydrophobic surface and interaction could come with a tradeoff of losing protein solubility [14], which in some cases may not be preferable for manufacturing water-soluble biosensor devices. Hydrogen bonds and salt bridges are weak electrostatic interactions of which the bond lengths are generally <3–4 Å for a weak interaction [15,16]. Although these bonds are generally weak, they are relatively stronger than hydrophobic interactions, and the accumulation of these bonds could yield considerable protein stability. A disulfide bond, however, is a strong covalent bond formed between sulfur atoms of two cysteine amino acids. Due to its high bonding energy, disulfide bonds can greatly enhance protein’s tolerance to extreme environments such as heat and acidity and can be found vastly in enzymes of thermophilic species [17,18,19]. The introduction of disulfide bonds for enhancing protein stability has been made to a number of enzymes, including formate dehydrogenase [20], T4 lysozyme [21], and bacterial alpha-type carbonic anhydrase [22].

Although adding disulfide bonds has been proven successful in modifying several enzymes, the modification of GOx by engineering disulfide bonds is yet to be investigated. To decipher the enzymatic structure and engineer the thermal stability of GOx, the rational exploration of the amino acid sequences should be realized. In this study, we used in silico approaches to predict possible mutations that can induce disulfide bonds and stabilize the structure of GOx. We systematically analyzed six PDB structures of GOx and highlighted potential amino acid residues that could be replaced by cysteine. Some of these residues were also shown to be less conserved and were confirmed to stabilize the protein upon cysteine mutations, making them promising candidates for future application of GOx. The engineered GOx could be of great benefit for broad applications in biosensor and bioelectronic industries and food and pharmaceutical sections.

## 2. Materials and Methods

### 2.1. Analysis of Disulfide Bonds

The initial Protein Data Bank (PDB) structure data set was retrieved from the MolProbity Top8000 database [23]. This database harbors 7957 high-quality and non-redundant PDB coordinates. We analyzed the characteristics of 2861 disulfide bonds detected in this data set by measuring S–S bond lengths and α angles (α1 is measured through Cβ1–Sγ1–Sγ2 and α2 through Sγ1–Sγ2–Cβ2) of all cysteine pairs that exhibit disulfide bonds. The average values for Cβ2–Cβ2 distance, S–S distance, and α angles (excluding outliers) were 3.85 Å, 2.10 Å, and 103.6°, respectively (see Supplementary File S1 for disulfide bond statistics). The characteristics of the disulfide bonds found in this data set were then used for screening for candidate residues of GOx that are suitable for mutagenesis.

### 2.2. Screening for Candidate Residues in GOx

Six PDB structures representing GOx from *A. niger*—3QVP (resolution: 1.20 Å), 3QVR (resolution: 1.30 Å), 5NIW (resolution: 1.80 Å), 5NIT (resolution: 1.87 Å), 1CF3 (resolution: 1.90 Å), and 1GAL (resolution: 2.30 Å)—were obtained from Protein Data Bank [24]. These are all *A. niger* GOx structures as reported in the UniProt database (accession number: P13006). The structures were screened for amino acid pairs that were 3.46–4.23 Å apart when measured for Cβ–Cβ distance. This range covers 95% of the disulfide bonds detected in the Top8000 database. In case of measuring a distance between residues that involves glycine, which does not have a Cβ atom, two virtual Cβ atoms were generated by replacing Hα2 and Hα3 atom of glycine with Cβ and extending the bond length to 1.53 Å (a typical Cα–Cβ length).

### 2.3. Cysteine Repacking and Assessment of Disulfide Bond

According to the Cβ–Cβ distance screening criteria, 118 amino acid pairs from the PDB structure 3QVP were identified as possible candidates for disulfide bond simulation. For each possible amino acid pair, the candidate residues were changed to cysteines by removing the side chains of amino acids that are within 5 Å away from the candidate residues and reintroducing side chains using SCWRL4 [25], keeping the protein backbone unaltered. This is also the same approach used in the Missense3D analysis pipeline [26].

Once the side chains were repacked, the mutated structures were analyzed for S–S bond length and α angles. Our analysis on disulfide bonds found in the Top8000 database suggested that, although the theoretical S–S bond length is 2.05 Å, the bond length found in PDB structures may vary due to minor errors in the coordinate files and the α angles can be flexible. To accommodate these uncertainties, we allowed acceptable bond length to be 1.54–2.67 Å and α angles to be 91.8–115.3 degrees. These ranges cover 95% of the data measured in the Top8000 database.

Generally, a mutation that results in a drastic change in cavity volume is likely to be deleterious to protein stability. Therefore, we further measured the change in total amino acid volumes upon cysteine mutations and screened out any candidate pairs that can, upon cysteine mutations, result in a drastic volume change of >70 Å, which is generally an upper limit of most observed cavities in protein [27].

### 2.4. Prediction of Functionally Important Residue and Mutation Effects on GOx

To determine functionally important residues, we used BLASTP [28] to find homologous sequences of *A. niger* GOx (UniProt accession number: P13006) against the SWISSPROT database. The sequences of the top 100 hits (all with E-value < 0.05) were then used in a multiple sequence alignment with MUSCLE (version 3.8.31) [29]. Next, the multiple sequence alignment profile was assessed for sequence conservation, and each amino acid residue was given a conservation score based on the Jensen–Shannon divergence algorithm [30]. The scores range from 0 to 1, with a higher number indicating greater conservation (Supplementary File S2).

To provide further details about mutation effects on a protein sequence, we used the SuSPect web server [31] for determining whether an amino acid residue might be prone to deleterious mutations or whether it might favor a cysteine mutation. Outputs from SuSPect range from 0 (neutral) to 100 (deleterious).

### 2.5. Prediction of Stability Changes on GOx upon Mutations

Stability changes of GOx structure upon mutations were assessed using DynaMut2 [32]. DynaMut2 is a web service that allows users to analyze changes in protein stability upon missense mutations based on changes in Gibbs free energy (ΔΔG), or the difference between the Gibbs free energy ΔG of the mutant structure and the ΔG of the wild-type structure. This tool has been shown to outperform many other tools on blind tests and also supports calculations of stability changes upon multiple mutations within the same structure.

## 3. Results and Discussion

The protein-based biocatalyst focused on by this study is a flavoprotein (130 to 175 kDa). The *A. niger* GOx is basically formed by two subunits (80 kDa each) [33]. These subunits are identical, consisting of a flavin adenine dinucleotide part and one iron. The flavin adenine dinucleotide cofactor (FAD/FADH_2_), when actively bound to GOx, can be found buried in the core of the enzyme (around 20 Å from the surface of GOx). This buried prosthetic group on each monomer is crucial for the reaction involving two protons and the transfer of two electrons from glucose.

**Figure 1:**
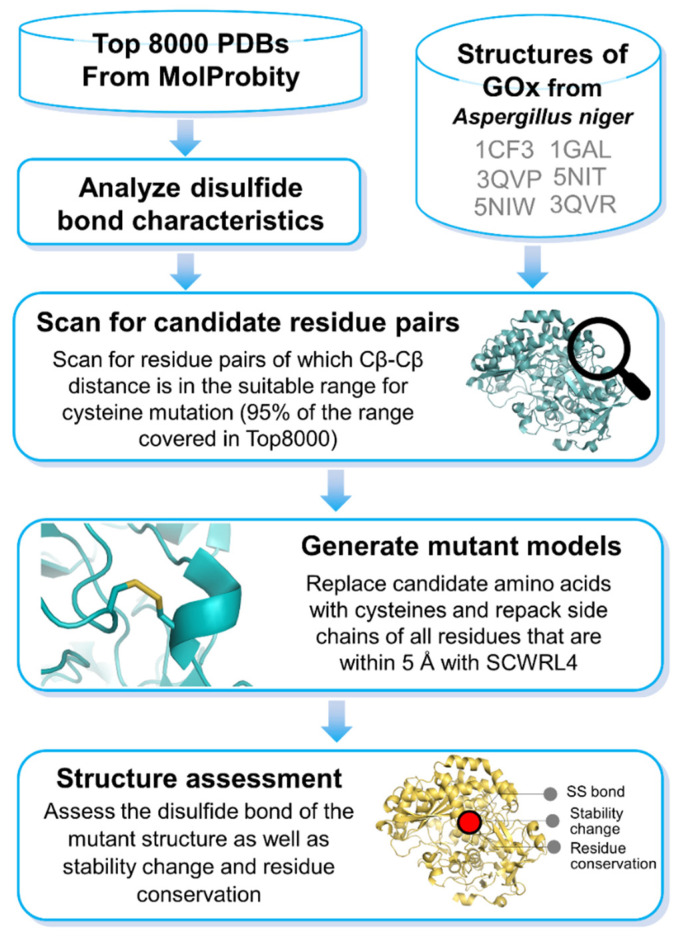
Conceptual illustration of the pipeline to realize rational bioengineering designs of GOx for optimizing protein stability.

**Figure 2:**
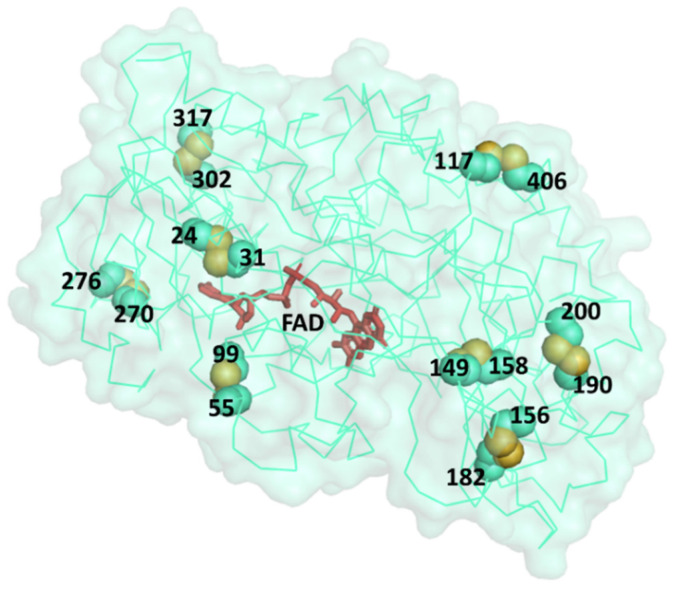
Three-dimensional (3D) structures of GOx (Cα trace and surface of the PDB structure 3QVP). Eight possible pairs for cysteine mutations are mapped onto this structure and are represented by spheres with sulfur atoms shown in yellow. Numbers shown in this figure indicate residue numbers according to the PDB coordinates. FAD (shown in red) can be found located at the pocket of the active site inside the protein. More details from different angles are shown in Supplementary Figure S1 (in Supplementary File S3) and Supplementary Video S1.

The structure of GOx originally has one disulfide bond linking together Cys164 and Cys206 as confirmed in all six PDB structures (which corresponds to Cys186–Cys228 in UniProt FASTA sequence). Upon screening for residue pairs in all six PDB coordinates according to our analysis pipeline (Figure 1), we found eight amino acid pairs suitable for cysteine mutation: Ile24–Gly31, Glu55–Gly99, Ala117–His406, Pro149–His158, Ala156–Tyr182, Met190–Thr200, Gly270–Thr276, and Gly302–Val317 (Figure 2 and Supplementary Video S1). Four of these residue pairs (Ile24–Gly31, Pro149–His158, Gly270–Thr276, and Gly302–Val317) were found in common in most GOx structures. Note that these residue numbers are according to their PDB coordinate system and are not the same as residue numbers in the GOx FASTA sequence. Details on Cβ–Cβ distances, S–S bond lengths, α angles, as well as predicted stability changes (ΔΔG) are shown in Table 1.

The calculation of ΔΔG using DynaMut2 confirms more stabilizing ΔΔG ranging from 0.13 to 0.99 kcal/mol in all Pro149 and His158 pairs detected across five PDB structures: 3QVP, 3QVR, 5NIW, 5NIT, and 1CF3. Additionally, Ala117 and His406, which were detected only in 1GAL, displayed stabilizing energy of 1.37 kcal/mol. Nonetheless, this amino acid pair yielded a relatively larger S–S distance (2.66 Å) than other candidate residue pairs.

Despite that the protein backbones of all the six PDB structures of GOx are highly similar and that they all shared 99–100% sequence identity (see Figures S2 and S3 in Supplementary File S3), the differences in numbers of detected candidate residue pairs could be due to some factors such as resolutions and atomic B-values (which were not assessed in this study). These factors could lead to minor differences in Cβ locations in 3D space, which, in turn, result in Cβ–Cβ distances greater than our defined threshold for disulfide bond candidacy.

**Figure 3:**
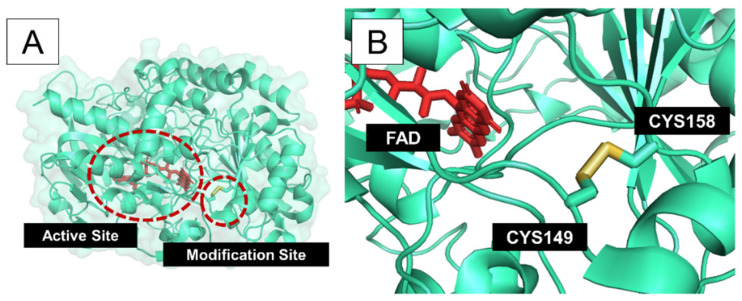
Predicted structure of mutated GOx with mutations Pro149Cys and His158Cys. (**A**) Overview of the location of the active site and modification site for Pro149Cys and His158Cys. (**B**) A closer look at the modification site. The disulfide bond is shown in yellow. FAD is shown in red.

Stability and activity are generally found to be in a tradeoff relationship [34]. This could be because some mutations are located near active sites or at functionally important residues. Therefore, we further assessed the conservation of the eight candidate residue pairs through Jensen–Shannon divergence scores (Table 2) and the effects of cysteine mutations with SuSPect (Table 3). It can be observed that both the conservation scores and the SuSPect prediction scores are broadly similar. The pair Ile24–Gly31 was shown by both analysis methods to be less suitable for mutations as both residues had high conservation scores and were more likely to be deleterious upon cysteine mutations. Interestingly, Pro149 and His158, which together have been previously shown to stabilize GOx upon cysteine mutations, were also shown to be less conserved, confirming their potential for disulfide bond modification. Upon closer inspection (Figure 2 and Figure 3), it can be seen that the residues 149 and 158 are located not too close to the active site where FAD binds to the protein, whereas the pair Ile24–Gly31 was located closer to the active site and could influence the enzymatic activity. This strongly explains why Ile24–Gly31 was predicted to be more conserved and likely to be deleterious and destabilizing upon cysteine mutations.

Some residue pairs showed contradictory results between sequence-based conservation analysis and structure-based stability analysis. For example, Gly302 was shown to be highly conserved when using Jensen–Shannon divergence scores, but results from SuSPect suggested that the mutation into cysteine could be likely neutral. Additional results from DynaMut2 suggested that the mutation of Gly302–Val317 to cysteines could be destabilizing, although the effect might not be as strong as when mutating Ile24–Gly31. It is common that inconsistency in the results can be observed when using multiple prediction tools. To overcome such a problem, one might suggest using more prediction tools and looking at the majority of the results to help verify the findings. Nevertheless, the best measure to cope with this situation is still to conduct laboratory experiments to verify the stability changes upon mutations.

Some residue pairs showed contradictory results between sequence-based conservation analysis and structure-based stability analysis. Some might suggest using multiple prediction tools to help verify the findings. Nevertheless, the best measure to cope with this situation is still to conduct laboratory experiments to verify the stability changes upon mutations.

A previous study by Broom et al. suggested that stabilizing mutations tend to increase hydrophobicity and that many approaches to enhance protein stability tend to follow this concept [14]. However, it was also shown that introducing hydrophobic residues can have an effect on reduced solubility. Rather than replacing surface residues with hydrophobic amino acids, this study uses an alternative approach to replace amino acids within suitable proximity to establish a disulfide bond, regardless of whether they are on the surface or within the protein core. Although cysteine is not a strong hydrophobic amino acid but can be classified more toward the hydrophobic spectrum, some may consider it as a polar or slightly polar amino acid. Remarkably, two cysteines can become less hydrophobic when they form a disulfide bond compared to free cysteines [35]. Thus, it is less likely that replacing amino acids with cysteines that can form a disulfide bond will result in decreased solubility.

Most structure-based protein stability prediction tools have their accuracies of about 60–80% [14,36]. DynaMut2 is currently the only recent web service that allows users to analyze protein stability change upon multiple point mutations, while the majority of widely used web services for protein stability assessment can only analyze single point mutations and/or are not available as web-based services [37], and are deemed unsuitable for assessing protein stability change upon disulfide formation where two amino acids are to be replaced with cysteines. Nevertheless, one study showed that DynaMut predictions yield a high false-positive rate (i.e., % of destabilizing mutations that were wrongly predicted to be stabilizing) as well as a high true-positive rate (i.e., % of stabilizing mutations that were correctly predicted to be stabilizing) [38]. To avoid false predictions, one might use multiple tools for protein assessment, which can be useful in helping researchers draw a conclusion based on results from several predictors.

Using in silico approaches to determine mutation sites for creating disulfide bonds is a great strategy for pre-screening possible candidate residues prior to a laboratory experiment. However, there are a limited number of available tools to help perform such an analysis [39,40,41]. Moreover, most of them rely mainly on measuring Cα–Cα distances, Cβ–Cβ distances, and bond angles, without assessing structural or functional impacts of cysteine mutations. Interestingly, a more recent web-based tool for disulfide bond engineering, Yosshi, allows for protein backbone adjustment, which might enable more residues pairs to be detected as potential candidates for disulfide modification [42]. However, it can be questioned whether altering protein backbones will affect protein activity. Our analysis approach in this GOx study is based on the concept of having the protein backbone fixed to minimize the chance of conformation change resulting in functional alteration. We also incorporate sequence-based and structure-based mutation analyses to ensure that the enzymatic activity of the engineered GOx is not interrupted.

One important limitation of this study is that it was performed only in silico. Although many computational results yielded by this study could support the possibility of mutating amino acid residues to cysteines for enhancing protein stability, the gold standard of proving such stability is to conduct site-directed mutagenesis, followed by protein expression and purification before determining enzyme activity and thermal stability of the mutated proteins through protein assays [20]. Verification of disulfide bond formation can be conducted using the SDS-PAGE technique [43] or using dithionitrobenzoic acid (DTNB) for detecting free thiol content as a result of having free cysteines [44].

Despite the necessity to verify the results of in silico protein engineering through wet-lab experiments, in silico modification of enzymes is a common approach that can be of great benefit for saving cost and time required for mutagenesis study and protein design as it can be a powerful tool for screening prior to laboratory experiments for GOx and other enzymes. It is envisioned that the findings in this study would represent an innovative and useful toolbox for bioengineering protein biocatalysts, opening opportunities for future research to explore robust enzymes for various applications, including biosensors, bioelectronics, and food and pharmaceutical sections

## 4. Conclusions

We have thoroughly investigated all the available enzymatic structures of GOx from *A. niger* and highlighted potential residues Pro149 and His158 for cysteine mutations that could yield disulfide bonds and provide more structural stability. Our in silico analysis suggested that cysteine mutations on proposed residues were less likely to interrupt protein functions. Unlike other engineering approaches, which tend to favor adding hydrophobic amino acids, sacrificing protein solubility for more thermal stability, the addition of disulfide bonds is less likely to lead to the solubility problem. This alternative technique of GOx structure modification by adding disulfide bond also has the potential to be applied in other protein engineering applications, allowing researchers to tailor robust enzymes for a wide variety of applications in the future. Further efforts should be made by investigating the in vitro effect of cysteine mutagenesis on stability and enzymatic reactivity in wet laboratories.

## Figures and Tables

**Table 1:** Candidate residue pairs for cysteine mutations. Residue numbers are based on PDB coordinates. Mutation pairs that were predicted stabilizing (positive ΔΔG) are highlighted in green.

PDB	Resolution (Å)	Residue 1 (PDB)	Residue 2 (PDB)	Cβ–Cβ (Å)	S–S (Å)	α1 (°)	α2 (°)	ΔΔG (kcal/mol)
3QVP	1.20	Ile24	Gly31	4.22	2.25	110.1	99.8	−2.31
		**Pro149**	**His158**	**3.84**	**2.28**	**111.9**	**103.3**	**0.96**
		Gly270	Thr276	3.68	2.19	108.5	113.7	−2.59
3QVR	1.30	Glu55	Gly99	4.19	2.48	93.0	99.2	−2.91
		**Pro149**	**His158**	**3.86**	**2.27**	**113.1**	**103.4**	**0.13**
		Gly270	Thr276	3.84	2.49	114.8	104.9	−2.61
		Gly302	Val317	3.66	2.22	110.0	93.1	−1.13
5NIW	1.80	Ile24	Gly31	4.21	2.61	113.7	99.2	−2.33
		**Pro149**	**His158**	**3.83**	**2.31**	**106.1**	**102.5**	**0.14**
		Gly270	Thr276	3.67	2.40	108.9	109.7	−2.62
		Gly302	Val317	3.69	2.27	109.6	97.9	−1.12
5NIT	1.87	Ile24	Gly31	4.09	2.66	108.8	95.9	−2.33
		**Pro149**	**His158**	**3.90**	**2.37**	**109.9**	**102.8**	**0.14**
		Gly270	Thr276	3.62	2.48	98.2	113.2	−2.62
		Gly302	Val317	3.69	2.14	109.6	96.2	−0.96
1CF3	1.90	**Pro149**	**His158**	**3.96**	**2.26**	**113.7**	**107.9**	**0.99**
		Gly270	Thr276	3.58	2.46	99.5	111.0	−2.65
1GAL	2.30	Ile24	Gly31	4.21	2.36	114.4	99.4	−2.33
		**Ala117**	**His406**	**3.88**	**2.66**	**98.7**	**113.6**	**1.37**
		Ala156	Tyr182	3.54	1.80	103.0	96.1	−2.04
		Met190	Thr200	4.15	2.08	105.3	93.4	−3.92

**Table 2:** The Jensen–Shannon divergence scores for each candidate residue pair. A higher score indicates greater residue conservation. (Residue numbers are according to the PDB 3QVP).

Residue 1	Conservation Score	Residue 2	Conservation Score
Ile24	0.66	Gly31	0.67
Glu55	0.38	Gly99	0.57
Ala117	0.43	His406	0.27
Pro149	0.29	His158	0.17
Ala156	0.29	Tyr182	0.29
Met190	0.33	Thr200	0.33
Gly270	0.28	Thr276	0.25
Gly302	0.72	Val317	0.51
**Color scale** least conserved: 0 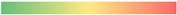 1: most conserved			

**Table 3:** SuSPect analysis of cysteine mutations on GOx sequence. (Residue numbers are according to the PDB 3QVP).

Residue 1	SuSPect Score	Residue 2	SuSPect Score
Ile24	79	Gly31	68
Glu55	37	Gly99	36
Ala117	14	His406	35
Pro149	55	His158	21
Ala156	10	Tyr182	31
Met190	17	Thr200	24
Gly270	24	Thr276	18
Gly302	17	Val317	44
**Color scale** predicted neutral: 0 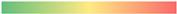 100: predicted deleterious			

## Data Availability

Data generated or analyzed during this study are included in this published article and its supplementary material files.

## Supplementary Materials

The following are available online at https://www.mdpi.com/article/10.3390/bioengineering8110188/s1, File S1: CA–CB distance. File S2: Conservation Score (3QVP), Conservation Score (all alignment), SuSPect Score. File S3: Figure S1: Different perspectives of three-dimensional (3D) structures of GOx (Cα trace and surface of the PDB structure 3QVP). Figure S2: Superposition of all PDB coordinates of all GOx used in this study. Figure S3: Multiple sequence alignment profile of six GOx structures. File S4: Video S1: 3D structure of GOx (3QVP) with eight candidate residue pairs for cysteine mutation.
