# Supplementary material for: In Silico Analysis of Glucose Oxidase from Aspergillus niger: Potential Cysteine Mutation Sites for Enhancing Protein Stability
Source: Bioengineering (Basel). 2021 Nov 19;8(11):188. doi: 10.3390/bioengineering8110188 (PMC8615187; doi:10.3390/bioengineering8110188)
Supplement: Supplementary file 1 [file bioengineering-08-00188-s001.zip › Supplementary File S3.pdf]

Supplementary Material

In-Silico Analysis of Glucose Oxidase from *Aspergillus niger*: Potential Cysteine Mutation Sites for Enhancing Protein Stability

Sirawit Ittisoponpisan[1*] and Itthipon Jeerapan[2,3,4*]

1. Center for Genomics and Bioinformatics Research, Division of Biological Science, Faculty of Science, Prince of Songkla University, Hat Yai, Songkhla 90110, Thailand
2. Center of Excellence for Trace Analysis and Biosensor, Prince of Songkla University, Hat Yai, Songkhla 90110, Thailand
3. Division of Physical Science, Faculty of Science, Prince of Songkla University, Hat Yai, Songkhla 90110, Thailand
4. Center of Excellence for Innovation in Chemistry, Faculty of Science, Prince of Songkla University, Hat Yai, Songkhla 90110, Thailand

***** Correspondence: sirawit.i@psu.ac.th (S.I.); itthipon.j@psu.ac.th (I.J.)

ORCID of Sirawit Ittisoponpisan: https://orcid.org/0000-0002-3340-0962
ORCID of Itthipon Jeerapan: https://orcid.org/0000-0001-8016-6411

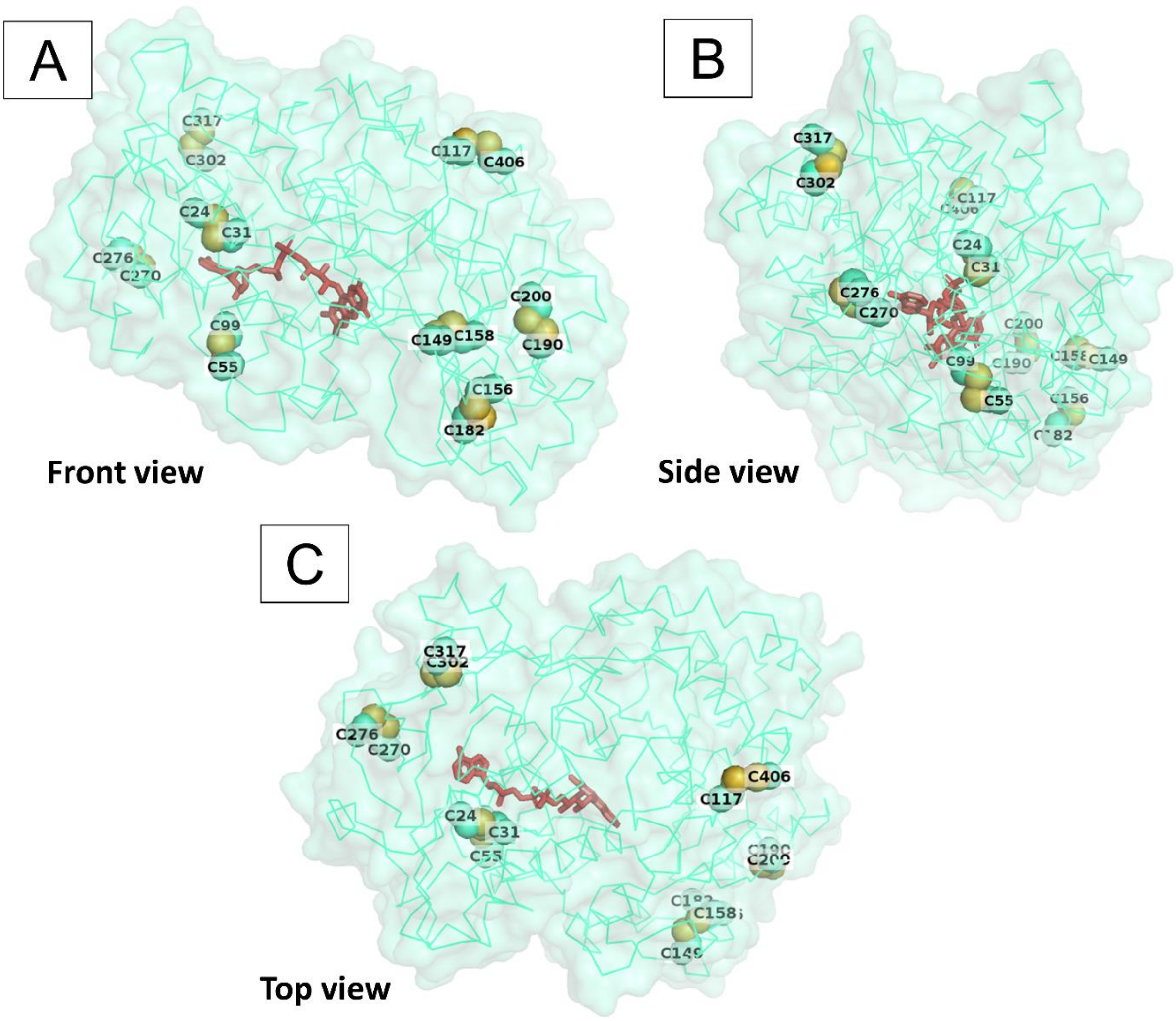

Figure S1. Different perspectives of three-dimensional (3D) structures of GOx (Cα trace and surface of the PDB structure 3QVP). The angles include A) front view, B) side view, and C) top view. Eight possible pairs for cysteine mutations are mapped onto this structure and are represented by spheres with sulfur atoms shown in yellow. Numbers shown in this figure indicate residue number according to the PDB coordinates. FAD (shown in red) can be found located at the pocket of the active site inside the protein.

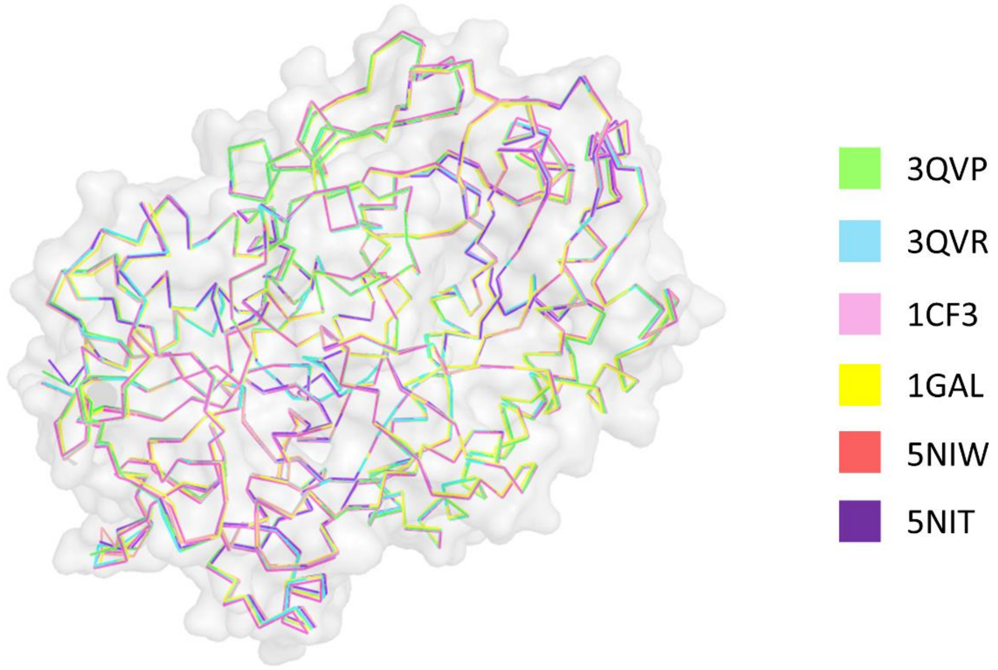

Figure S2. Superposition of all PDB coordinates of all GOx used in this study. The structures are displayed as Cα traces. To give a general outlook of GOx, the protein surface (based on PDB 3QVP) is shown in light grey.

```
3QVP_1|Chain      SNGIEASLLTDPKDVSGRTVDYIIAGGGLTGLTTAARLTENPNISVLVIESGSYESDRGP  60
3QVR_1|Chain      SNGIEASLLTDPKDVSGRTVDYIIAGGGLTGLTTAARLTENPNISVLVIESGSYESDRGP  60
1CF3_1|Chain      SNGIEASLLTDPKDVSGRTVDYIIAGGGLTGLTTAARLTENPNISVLVIESGSYESDRGP  60
1GAL_1|Chain      SNGIEASLLTDPKDVSGRTVDYIIAGGGLTGLTTAARLTENPNISVLVIESGSYESDRGP  60
5NIW_1|Chain      --GIEASLLTDPKDVSGRTVDYIIAGGGLVGLTTAAKLTENPNISVLVIESGSYESDRGP  58
5NIT_1|Chain      --GIEASLLTDPKDVSGRTVDYIIAGGGLVGLTTAARLTENPNISVLVIESGSYESDRGP  58
                    ***************************.******:***********************

3QVP_1|Chain      IIEDLNAYGDIFGSSVDHAYETVELATNNQTALIRSGNGLGGSTLVNGGTWTRPHKAQVD  120
3QVR_1|Chain      IIEDLNAYGDIFGSSVDHAYETVELATNNQTALIRSGNGLGGSTLVNGGTWTRPHKAQVD  120
1CF3_1|Chain      IIEDLNAYGDIFGSSVDHAYETVELATNNQTALIRSGNGLGGSTLVNGGTWTRPHKAQVD  120
1GAL_1|Chain      IIEDLNAYGDIFGSSVDHAYETVELATNNQTALIRSGNGLGGSTLVNGGTWTRPHKAQVD  120
5NIW_1|Chain      IIEDLNAYGDIFGSSVDHAYETVELATNNQTALVRSGNGLGGSTLINGGTWTRPHKAQVD  118
5NIT_1|Chain      IIEDLNAYGDIFGSSVDHAYETVELATNNQTALVRSGNGLGGSTLVNGGTWTRPHKAQVD  118
                  *********************************:***********:**************

3QVP_1|Chain      SWETVFGNEGWNWDNVAAYSLQAERARAPNAKQIAAGHYFNASCHGVNGTVHAGPRDTGD  180
3QVR_1|Chain      SWETVFGNEGWNWDNVAAYSLQAERARAPNAKQIAAGHYFNASCHGVNGTVHAGPRDTGD  180
1CF3_1|Chain      SWETVFGNEGWNWDNVAAYSLQAERARAPNAKQIAAGHYFNASCHGVNGTVHAGPRDTGD  180
1GAL_1|Chain      SWETVFGNEGWNWDNVAAYSLQAERARAPNAKQIAAGHYFNASCHGVNGTVHAGPRDTGD  180
5NIW_1|Chain      SWETVFGNEGWNWDNVAAYSLQAERARAPNAKQIAAGHYFNTSCHGVNGTVHAGPRDTGD  178
5NIT_1|Chain      SWETVFGNEGWNWDNVAAYSLQAERARAPNAKQIAAGHYFNTSCHGVNGTVHAGPRDTGD  178
                  *****************************************:******************

3QVP_1|Chain      DYSPIVKALMSAVEDRGVPTKKDFGCGDPHGVSMFPNTLHEDQVRSDAAREWLLPNYQRP  240
3QVR_1|Chain      DYSPIVKALMSAVEDRGVPTKKDFGCGDPHGVSMFPNTLHEDQVRSDAAREWLLPNYQRP  240
1CF3_1|Chain      DYSPIVKALMSAVEDRGVPTKKDFGCGDPHGVSMFPNTLHEDQVRSDAAREWLLPNYQRP  240
1GAL_1|Chain      DYSPIVKALMSAVEDRGVPTKKDFGCGDPHGVSMFPNTLHEDQVRSDAAREWLLPNYQRP  240
5NIW_1|Chain      DYSPIVKALMSAVEDRGVPTKKDFGCGDPHGVSMFPNTLHEDQVRSDAAREWLLPNYQRP  238
5NIT_1|Chain      DYSPIVKALMSAVEDRGVPTKKDFGCGDPHGVSMFPNTLHEDQVRSDAAREWLLPNYQRP  238
                  ************************************************************

3QVP_1|Chain      NLQVLTGQYVGKVLLSQNGTTPRAVGVEFGTHKGNTHNVYAKHEVLLAAGSAVSPTILEY  300
3QVR_1|Chain      NLQVLTGQYVGKVLLSQNGTTPRAVGVEFGTHKGNTHNVYAKHEVLLAAGSAVSPTILEY  300
1CF3_1|Chain      NLQVLTGQYVGKVLLSQNGTTPRAVGVEFGTHKGNTHNVYAKHEVLLAAGSAVSPTILEY  300
1GAL_1|Chain      NLQVLTGQYVGKVLLSQNGTTPRAVGVEFGTHKGNTHNVYAKHEVLLAAGSAVSPTILEY  300
5NIW_1|Chain      NLQVLTGQYVGKVLLSQNGTTPRAVGVEFGTHKGNTHNVYAKHEVLLAAGSAVSPTILEY  298
5NIT_1|Chain      NLQVLTGQYVGKVLLSQNGTTPRAVGVEFGTHKGNTHNVYAKHEVLLAAGSAVSPTILEY  298
                  ************************************************************

3QVP_1|Chain      SGIGMKSILEPLGIDTVVDLPVGLNLQDQTTATVRSRITSAGAGQGQAAWFATFNETFGD  360
3QVR_1|Chain      SGIGMKSILEPLGIDTVVDLPVGLNLQDQTTATVRSRITSAGAGQGQAAWFATFNETFGD  360
1CF3_1|Chain      SGIGMKSILEPLGIDTVVDLPVGLNLQDQTTATVRSRITSAGAGQGQAAWFATFNETFGD  360
1GAL_1|Chain      SGIGMKSILEPLGIDTVVDLPVGLNLQDQTTATVRSRITSAGAGQGQAAWFATFNETFGD  360
5NIW_1|Chain      SGIGMKSILEPLGIDTVVDLPVGLNLQDQTTATVRSRITSAGAGQGQAAWFATFNETFGD  358
5NIT_1|Chain      SGIGMKSILEPLGIDTVVDLPVGLNLQDQTTATVRSRITSAGAGQGQAAWFATFNETFGD  358
                  ************************************************************

3QVP_1|Chain      YSEKAHELLNTKLEQWAEEAVARGGFHNTTALLIQYENYRDWIVNHNVAYSELFLDTAGV  420
3QVR_1|Chain      YSEKAHELLNTKLEQWAEEAVARGGFHNTTALLIQYENYRDWIVNHNVAYSELFLDTAGV  420
1CF3_1|Chain      YSEKAHELLNTKLEQWAEEAVARGGFHNTTALLIQYENYRDWIVNHNVAYSELFLDTAGV  420
1GAL_1|Chain      YSEKAHELLNTKLEQWAEEAVARGGFHNTTALLIQYENYRDWIVNHNVAYSELFLDTAGV  420
5NIW_1|Chain      YSEKAHELLNTKLEQWAEEAVARGGFHNTTALLIQYENYRDWIVNHNVAYSELFLDTAGV  418
5NIT_1|Chain      YSEKAHELLNTKLEQWAEEAVARGGFHNTTALLIQYENYRDWIVNHNVAYSELFLDTAGV  418
                  ************************************************************

3QVP_1|Chain      ASFDVWDLLPFTRGYVHILDKDPYLHHFAYDPQYFLNELDLLGQAAATQLARNISNSGAM  480
3QVR_1|Chain      ASFDVWDLLPFTRGYVHILDKDPYLHHFAYDPQYFLNELDLLGQAAATQLARNISNSGAM  480
1CF3_1|Chain      ASFDVWDLLPFTRGYVHILDKDPYLHHFAYDPQYFLNELDLLGQAAATQLARNISNSGAM  480
1GAL_1|Chain      ASFDVWDLLPFTRGYVHILDKDPYLHHFAYDPQYFLNELDLLGQAAATQLARNISNSGAM  480
5NIW_1|Chain      ASFDVWDLLPFTRGYVHILDKDPYLHHFAYDPQYFLNELDLLGQAAATQLARNISNSGAM  478
5NIT_1|Chain      ASFDVWDLLPFTRGYVHILDKDPYLHHFAYDPQYFLNELDLLGQAAATQLARNISNSGAM  478
                  ************************************************************

3QVP_1|Chain      QTYFAGETIPGDNLAYDADLSAWTEYIPYHFRPNYHGVGTCSMMPKEMGGVVDNAARVYG  540
3QVR_1|Chain      QTYFAGETIPGDNLAYDADLSAWTEYIPYHFRPNYHGVGTCSMMPKEMGGVVDNAARVYG  540
1CF3_1|Chain      QTYFAGETIPGDNLAYDADLSAWTEYIPYHFRPNYHGVGTCSMMPKEMGGVVDNAARVYG  540
1GAL_1|Chain      QTYFAGETIPGDNLAYDADLSAWTEYIPYHFRPNYHGVGTCSMMPKEMGGVVDNAARVYG  540
5NIW_1|Chain      QTYFAGETIPGDNLAYDADLSAWTEYIPYHFRPNYHGVGTCSMMPKEMGGVVDNAARVYG  538
5NIT_1|Chain      QTYFAGETIPGDNLAYDADLSAWTEYIPYHFRPNYHGVGTCSMMPKEMGGVVDNAAKVYG  538
                  ********************************************************:***

3QVP_1|Chain      VQGLRVIDGSIPPTQMSSHVMTVFYAMALKISDAILEDYASMQ    583
3QVR_1|Chain      VQGLRVIDGSIPPTQMSSHVMTVFYAMALKISDAILEDYASMQ    583
1CF3_1|Chain      VQGLRVIDGSIPPTQMSSHVMTVFYAMALKISDAILEDYASMQ    583
1GAL_1|Chain      VQGLRVIDGSIPPTQMSSHVMTVFYAMALKISDAILEDYASMQ    583
5NIW_1|Chain      VQGLRVIDGSIPPTQVSSHVMTVFYAMALKISDAILEDYASMQ    581
5NIT_1|Chain      VQGLRVIDGSIPPTQVSSHVMTVFYAMALKISDAILEDYASMQ    581
                  ***************:***************************
```

Figure S3. Multiple sequence alignment profile of six GOx structures. Amino acid variants are highlighted in yellow.
